# First Genome Sequence of Potential Mycotoxin-Degrading Bacterium *Devosia nanyangense* DDB001

**DOI:** 10.1128/genomeA.00922-14

**Published:** 2014-10-23

**Authors:** Mark Onyango, Yang Wang, Ole Nickel, Chen Zhao, Xiaolin Zhang, Axel Hartke, Juergen Hemberger, Franz Cemic

**Affiliations:** a Institute for Biochemical Engineering & Analytics, University of Applied Sciences Giessen, Giessen, Germany; b Academy of State Administration of Grain, Beijing, PR China; c Université de Caen Basse-Normandie, IBFA, U2RM Stress Virulence, Caen, France

## Abstract

*Devosia* sp. nov. DDB001, isolated from mycotoxin-contaminated soil, is a potential mycotoxin-degrading alphaproteobacterium. To our knowledge, this is the first draft genome announcement of a *Devosia* species.

## GENOME ANNOUNCEMENT

The genus *Devosia* is a member of the *Alphaproteobacteria* and comprises Gram-negative, rod-shaped, non-spore-forming species. The genus *Devosia* was created by the reclassification of *Pseudomonas riboflavina* as *Devosia riboflavin* (1). At the time of this writing, 15 culturable species with validated names have been described (2), but no genome sequence of a *Devosia* species had been published until now. We present here the first draft genome sequence of a species of the genus *Devosia*. The sequenced strain, isolated in Nanyang, China, seems to correspond to a novel species for which the name *Devosia nanyangense* sp. nov. is proposed. The strain was isolated from screening of mycotoxin-contaminated soil.

A bacterial culture that degrades mycotoxin deoxynivalenol (DON) was obtained from soil samples collected in wheat fields of Nanyang, China, using a continuous enrichment culture procedure. The isolated bacterium, designated strain DDB001, completely removed 200 µg/ml of DON from the culture medium after incubation for 3 days. The isolate still showed full activity to transform DON after 10 consequent subcultures, proving that the degradation capability of the selected isolate is consistent. The physiological, biochemical, and phylogenetic properties of strain DDB001 show that DDB001 was classified as a novel species within the genus *Devosia*, for which the name *Devosia nanyangense* sp. nov. is proposed. High-performance liquid chromatography-mass spectrometry analysis indicated that a metabolite of DON has the same molecular weight as that of DON, but has slightly different chromatographic behavior. We are continuing to study the chemical structure of this metabolite.

The whole-genome sequencing of *Devosia nanyangense* sp. nov. was performed using an Illumina HighSeq2000 Sequencer at BGI (http://www.genomics.cn/en) in Shenzhen. The 90-bp paired-end reads were trimmed and assembled *de novo* using Velvet 1/2/08 (3). The genome annotation was performed by the RAST annotation service (version 4.0) (http://rast.nmpdr.org) (4). The draft genome sequence of *Devosia* sp. nov. DDB001 consists of 4.669.456 bp (average depth of coverage, 106,077 bp) contained in 95 contigs with an average GC content of 64%. The *N*_50_ contig was approximately 109 kb, and the largest contig was 283 kb. A total of 4,307 open reading frames (ORFs), 47 tRNAs, and 3 rRNAs were identified in the draft genome.

### Nucleotide sequence accession numbers.

The results of this whole-genome shotgun project have been deposited at the European Nucleotide Archive under the accession numbers CCAO010000001 through CCAO010000095.
